# Renal replacement therapy is independently associated with a lower risk of death in patients with severe acute kidney injury treated with targeted temperature management after out-of-hospital cardiac arrest

**DOI:** 10.1186/s13054-020-2822-x

**Published:** 2020-03-23

**Authors:** Yoon Hee Choi, Dong Hoon Lee, Je Hyeok Oh, Jung Hee Wee, Tae Chang Jang, Seung Pill Choi, Kyu Nam Park, on behalf of the Korean Hypothermia Network Investigators, Seung Pill Choi, Kyu Nam Park, Minjung Kathy Chae, Won Young Kim, Byung Kook Lee, Dong Hoon Lee, Tae Chang Jang, Jae Hoon Lee, Yoon Hee Choi, Je Sung You, Young Hwan Lee, In Soo Cho, Su Jin Kim, Jong-Seok Lee, Yong Hwan Kim, Min Seob Sim, Jonghwan Shin, Yoo Seok Park, Hyung Jun Moon, Won Jung Jeong, Joo Suk Oh, Kyoung-Chul Cha

**Affiliations:** 1 Department of Emergency Medicine, Ewha Womans University Medical Center and Ewha Womans University Mokdong Hospital, 1071, Anyangcheon-ro, Yangcheon-gu, Seoul, 07985 Republic of Korea; 2 Department of Emergency Medicine, Chung-Ang University College of Medicine, 84, Heukseok-ro, Dongjak-gu, Seoul, 06974 Republic of Korea; 3 Department of Emergency Medicine, Wonkwang University College of Medicine, Sanbon Hospital, 321, Snabon-ro, Gunpo-si, Gyeonggi-do, 15865 Republic of Korea; 4 Department of Emergency Medicine, Daegu Catholic University School of Medicine, 33, Duryugongwon-ro 17-gil, Nam-gu, Daegu, 42472 Republic of Korea; 5 Department of Emergency Medicine, Eunpyeong St. Mary’s Hospital, The Catholic University of Korea College of Medicine, 1021, Tongil-ro, Eunpyeong-gu, Seoul, 03312 Republic of Korea; 6 Department of Emergency Medicine, Seoul St. Mary’s Hospital, The Catholic University of Korea College of Medicine, 222, Banpo-daero, Seocho-gu, Seoul, 06591 Republic of Korea

**Keywords:** Renal replacement therapy, Acute kidney injury, Out-of-hospital cardiac arrest, Targeted temperature management, Therapeutic hypothermia

## Abstract

**Background:**

The effect of renal replacement therapy (RRT) on the outcomes of severe acute kidney injury (AKI) after out-of-hospital cardiac arrest (OHCA) is uncertain. This study aimed to evaluate the association of RRT with 6-month mortality in patients with severe AKI treated with targeted temperature management (TTM) after OHCA.

**Methods:**

This was a retrospective analysis of a prospectively collected multicentre observational cohort study that included adult OHCA patients treated with TTM across 22 hospitals in South Korea between October 2015 and December 2018. AKI was diagnosed using the Kidney Disease: Improving Global Outcomes criteria. The primary outcome was 6-month mortality and the secondary outcome was cerebral performance category (CPC) at 6 months. Multivariate Cox regression analysis was performed to define the role of RRT in stage 3 AKI.

**Results:**

Among 10,426 patients with OHCA, 1373 were treated with TTM. After excluding those who died within 48 h of return of spontaneous circulation (ROSC) and those with pre-arrest chronic kidney disease, our study cohort comprised 1063 patients. AKI developed in 590 (55.5%) patients and 223 (21.0%) had stage 3 AKI. Among them, 115 (51.6%) were treated with RRT. The most common treatment modality among RRT patients was continuous renal replacement therapy (111 [96.5%]), followed by intermittent haemodialysis (4 [3.5%]). The distributions of CPC (1–5) at 6 months for the non-RRT vs. the RRT group were 3/108 (2.8%) vs. 12/115 (10.4%) for CPC 1, 0/108 (0.0%) vs. 1/115 (0.9%) for CPC 2, 1/108 (0.9%) vs. 3/115 (2.6%) for CPC 3, 6/108 (5.6%) vs. 6/115 (5.2%) for CPC 4, and 98/108 (90.7%) vs. 93/115 (80.9%) for CPC 5, respectively (*P* = 0.01). The RRT group had significantly lower 6-month mortality than the non-RRT group (93/115 [81%] vs. 98/108 [91%], *P* = 0.04). Multivariate Cox regression analyses showed that RRT was independently associated with a lower risk of death in patients with stage 3 AKI (hazard ratio, 0.569 [95% confidence interval, 0.377–0.857, *P* = 0.01]).

**Conclusion:**

Dialysis interventions were independently associated with a lower risk of death in patients with stage 3 AKI treated with TTM after OHCA.

## Background

Acute kidney injury (AKI) develops frequently after out-of-hospital cardiac arrest (OHCA) and is associated with long-term mortality and poor neurological outcomes [1–8]. Similar to critically ill patients, increased AKI severity is associated with increased mortality in OHCA patients [2, 4, 9]. In contrast, recovery from AKI was a potent predictor of survival and good neurological outcome at discharge [5]. Mortality rates of patients with stage 3 AKI after OHCA are reported to be over 70% [2, 3]. Although renal replacement therapy (RRT) is a treatment option for these patients, its impact on outcomes is uncertain. We hypothesised that applying RRT would be associated with better outcomes in patients who develop severe AKI after OHCA.

Two recent studies evaluated the effect of RRT on cardiac arrest outcomes [10, 11]. However, these studies did not show a risk reduction in patients receiving RRT. On the contrary, one of them reported a significant increase in mortality among patients receiving RRT [11]. The two studies did not consider AKI stage in their comparison of outcomes in patients with and without RRT. Because all patients who received RRT had stage 3 AKI, the risk of death in the RRT group was higher than that in the non-RRT group. Therefore, this study evaluated whether dialysis interventions had an impact on the outcomes of patients with stage 3 AKI after OHCA who were treated with targeted temperature management (TTM).

## Material and methods

### Study design and setting

This was a retrospective analysis of the prospectively collected multicentre observational cohort study. Data were collected from the Korean Hypothermia Network (KORHN) prospective registry (KORHN-PRO) between October 2015 and December 2018. The KORHN is a multicentre clinical research consortium for TTM in South Korea. In total, 22 academic hospitals participated in the KORHN-PRO.

### Study population

All adult patients (≥ 18 years) with OHCA, regardless of the cause of arrest, who were unconscious (Glasgow Coma Scale score < 8) after the return of spontaneous circulation (ROSC) and treated with TTM, were enrolled in the registry. Patients with active intracranial bleeding, acute stroke, known limitations in therapy and do-not-attempt resuscitation order, known pre-arrest cerebral performance category (CPC) 3 or 4, known disease making 6-month survival unlikely, and body temperature < 30 °C on admission were excluded.

Enrolled patients received care for post-cardiac arrest syndrome (PCAS) according to standard operating procedures regarding OHCA at each hospital. The principal investigator of each participating hospital collected data from the hospital records of OHCA survivors treated with TTM. Using a telephone survey, the independent data input committee investigated the survival and neurological status 6 months after ROSC. If patients died before 6 months, the death date was recorded.

### Variables

The primary outcome was 6-month mortality and the secondary outcome was CPC at 6 months. We examined the following variables: age, sex, body weight, medical history (hypertension, heart failure, diabetes mellitus), arrest location (home, workplace, street, public building, nursing home, unknown), witnessed arrest, bystander cardiopulmonary resuscitation (CPR), time from collapse to CPR, time from CPR to ROSC, epinephrine dose, initial rhythm assessed by emergency medical service personnel in the field (ventricular fibrillation, pulseless ventricular tachycardia, pulseless electrical activity, asystole, unknown shockable, unknown non-shockable, unknown), arrest cause (medical or unknown, trauma, submersion, drug overdose, asphyxia, hanging), post-ROSC lactate, post-ROSC shock (cardiovascular sequential organ failure assessment score ≥ 1 on day 1), targeted temperature of TTM (33 or 36 °C), duration of TTM (24 or 48 h), adverse events during the 7 days following ROSC (seizure, bleeding, sepsis, hypoglycaemia [blood glucose< 60 mg/dl], sustained hyperglycaemia [blood glucose > 180 mg/dl for > 4 h], tachycardia [> 130/min], and bradycardia [< 40/min]), RRT modality (continuous renal replacement therapy [CRRT] or intermittent haemodialysis), RRT initiation time, reasons for initiating/terminating RRT, dialysis dependence at discharge, and serum creatinine (SCr). The KORHN-PRO required input of data on SCr daily until 7 days after ROSC. If the SCr was checked twice or more in 1 day, the highest value was collected in the registry.

### Definition of AKI

We defined AKI based on the diagnostic criteria stipulated in the Kidney Disease: Improving Global Outcomes (KDIGO) guidelines [12]. Because we did not have data on patients’ prior kidney function, we used the “modification of diet in renal disease study” formula for glomerular filtration rate (GFR) to estimate baseline SCr using the lower end of the normal range of GFR [13]. After estimating baseline SCr, we determined whether the patients had an AKI and subsequently classified them into AKI stages. A patient was defined as having AKI if one of the SCr measurements during the 7-day period was ≥ 150% of the baseline SCr or if the SCr increased ≥ 0.3 mg/dl within a 48-h interval during the 7-day period. Once AKI status was determined, AKI staging was performed. A 150–199%, 200–299%, and ≥ 300% increase from baseline SCr in one of the SCr measurements during the 7-day period was defined as stage 1, 2, and 3 AKI, respectively. Regardless of fulfilling these criteria, patients were defined as developing stage 3 AKI when SCr increased ≥ 4.0 mg/dl or RRT was initiated. We could not use the criteria for hourly urine output because data on hourly urine output were not collected in the registry.

### Statistical methods

Descriptive statistics are reported as median (interquartile range) or mean ± standard deviation for continuous variables according to the normality of distribution. Data normality was analysed using Shapiro–Wilk test or Kolmogorov–Smirnov test. Categorical variables are reported as frequency (percentage). Demographics and clinical differences between groups were assessed using Pearson’s chi-squared test, Fisher’s exact test, independent sample *t*-test, or Mann-Whitney *U* test, as appropriate. We performed multivariate Cox regression analysis to calculate the hazard ratio (HR) and 95% confidence interval (CI) of RRT with respect to mortality and to find independent factors associated with risk of death. All variables were entered into a multivariate Cox regression analysis. Differences in Kaplan-Meier survival curves between RRT and non-RRT groups were compared using the log-rank test. A *P* value < 01.05 was considered statistically significant. Statistical analyses were performed using IBM SPSS version 25.0 (IBM Corp., Armonk, NY, USA).

## Results

### Study population

**Fig. 1:**
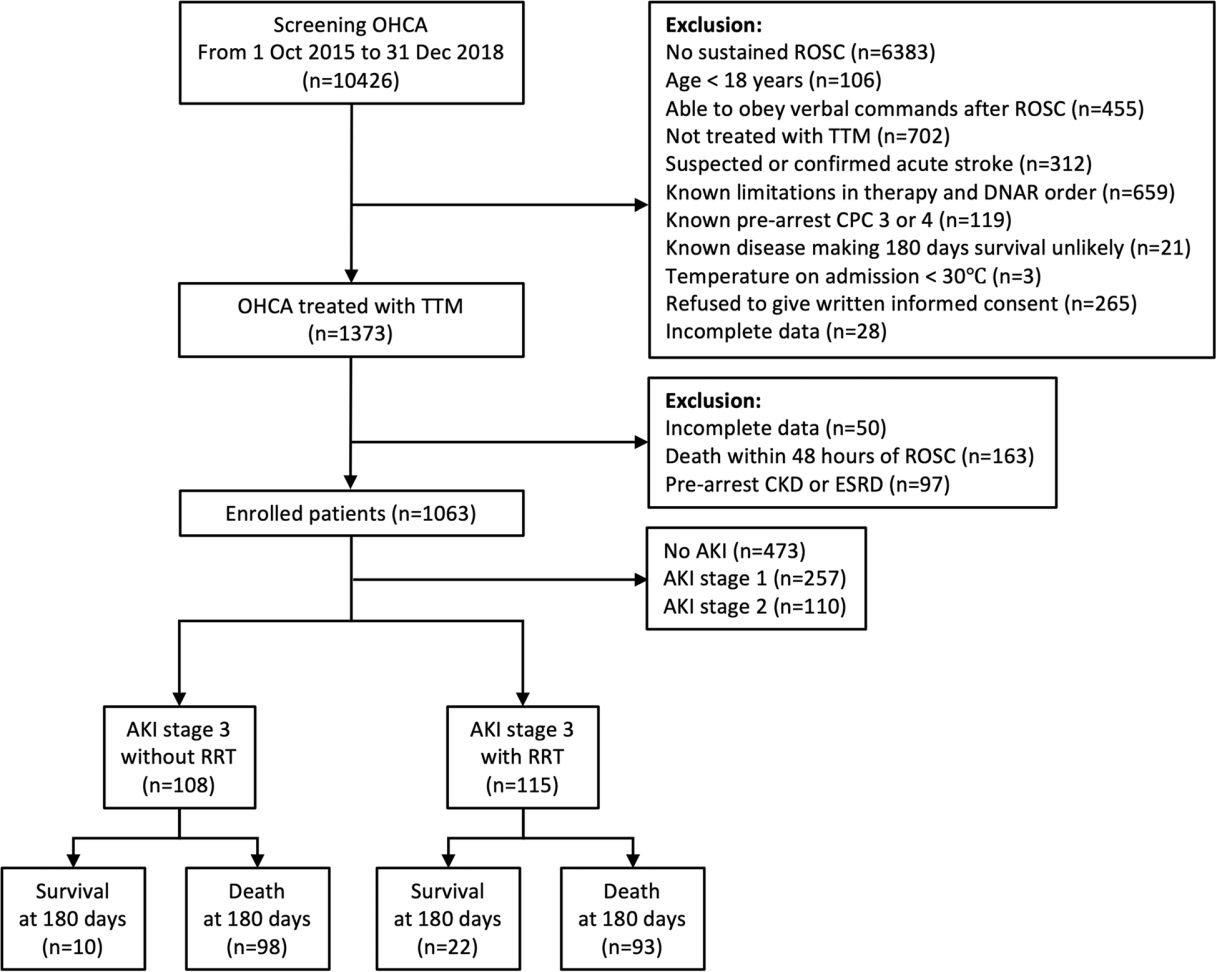
Study flow chart. AKI, acute kidney injury; CKD, chronic kidney disease; CPC, cerebral performance category; DNAR, do-not-attempt resuscitation; ESRD, end-stage renal disease; OHCA, out-of-hospital cardiac arrest; ROSC, return of spontaneous circulation; RRT, renal replacement therapy; TTM, targeted temperature management

Out of 10,426 patients with OHCA that were screened during the study period, 1373 were treated with TTM at 22 academic hospitals throughout South Korea (Fig. 1). We excluded the patients who died within 48 h of ROSC to minimise competing risk, those with pre-arrest chronic kidney disease or end-stage renal disease, and those with incomplete data such as SCr, survival, or neurological status at 6 months. In total, 1063 patients were enrolled in the study cohort. The baseline characteristics of stage 3 patients with AKI according to RRT status and outcomes are summarised in Tables 1 and 2.

**Table 1:** Patient characteristics according to the status of renal replacement therapy in patients who developed stage 3 acute kidney injury after out-of-hospital cardiac arrest

	Total (*n* = 223)	Non-RRT group (*n* = 108)	RRT group (*n* = 115)	*P* value
**Baseline characteristics**				
Age, years	61 (49–73)	64 (52–73)	61 (49–73)	0.39
Male gender	156 (70.3)	71 (65.7)	85 (74.6)	0.15
Body weight, kg	69 (60–75)	65 (59–73)	69 (60–75)	0.16
**Past medical history**				
Hypertension	104 (46.6)	55 (50.9)	49 (42.6)	0.21
Heart failure	10 (4.5)	4 (3.7)	6 (5.2)	0.75
Diabetes mellitus	65 (29.1)	35 (32.4)	30 (26.1)	0.30
**Cardiac arrest**				
Arrest location				0.09
Home	126 (56.5)	67 (62.0)	59 (51.3)	
Workplace	10 (4.5)	4 (3.7)	6 (5.2)	
Street	47 (21.1)	19 (17.6)	28 (24.3)	
Public building	30 (13.5)	16 (14.8)	14 (12.2)	
Nursing home	5 (2.2)	1 (0.9)	4 (3.5)	
Unknown	5 (2.2)	1 (0.9)	4 (3.5)	
Witnessed arrest	138 (62.7)	62 (58.5)	76 (66.7)	0.21
Bystander CPR	136 (62.1)	64 (59.8)	72 (64.3)	0.50
Time from collapse to CPR, min	1 (0–7)	3 (0–8)	1 (0–7)	0.58
Time from CPR to ROSC, min	40 (26–53)	40 (29–50)	40 (26–53)	0.75
Epinephrine, mg	2 (1–7)	3 (1–5)	2 (1–7)	0.83
Initial rhythm				**0.04**
Vf	48 (21.5)	12 (11.1)	36 (31.3)	
Pulseless VT	1 (0.4)	1 (0.9)	0 (0.0)	
PEA	47 (21.1)	24 (22.2)	23 (20.0)	
Asystole	91 (40.8)	55 (50.9)	36 (31.3)	
Unknown shockable	4 (1.8)	2 (1.9)	2 (1.7)	
Unknown non-shockable	1 (0.4)	0 (0.0)	1 (0.9)	
Unknown	31 (13.9)	14 (13.0)	17 (14.8)	
Arrest cause				**< 0.001**
Medical or unknown	168 (75.3)	68 (63.0)	100 (87.0)	
Trauma	5 (2.2)	3 (2.8)	2 (1.7)	
Submersion	5 (2.2)	4 (3.7)	1 (0.9)	
Drug overdose	3 (1.3)	1 (0.9)	2 (1.7)	
Asphyxia	12 (5.4)	10 (9.3)	2 (1.7)	
Hanging	30 (13.5)	22 (20.4)	8 (7.0)	
**Post-resuscitation**				
Lactate after ROSC, mmol/L	12.2 (8.9–14.8)	11.4 (8.6–14.5)	12.2 (8.9–14.8)	0.36
Shock after ROSC	210 (94.2)	101 (93.5)	109 (94.8)	0.69
TT of 36 °C	47 (21.1)	18 (16.7)	29 (25.2)	0.12
TTM duration of 48 h	5 (2.2)	3 (2.8)	2 (1.7)	0.68
**Adverse events during 7 days since ROSC**				
Seizure	38 (17.0)	17 (15.7)	21 (18.3)	0.62
Bleeding	16 (7.2)	3 (2.8)	13 (11.3)	**0.02**
Sepsis	54 (24.2)	24 (22.2)	30 (26.1)	0.50
Hypoglycaemia	44 (19.7)	18 (16.7)	26 (22.6)	0.27
Sustained hyperglycaemia	153 (68.6)	72 (66.7)	81 (70.4)	0.55
Tachycardia	49 (22.1)	24 (22.2)	25 (21.9)	0.96
Bradycardia	6 (2.7)	3 (2.8)	3 (2.6)	1.00
Re-arrest	55 (24.8)	22 (20.6)	34 (28.7)	0.16

Values are expressed as number (%) or median (interquartile range)

*P* < 0.05 are presented in bold

*CPR* cardiopulmonary resuscitation, *PEA* pulseless electrical activity, *ROSC* return of spontaneous circulation, *RRT* renal replacement therapy, *TT* target temperature, *TTM* targeted temperature management, *Vf* ventricular fibrillation, *VT* ventricular tachycardia

**Table 2:** Outcomes according to the status of renal replacement therapy in patients who developed stage 3 acute kidney injury after out-of-hospital cardiac arrest

	Total (*n* = 223)	Non-RRT group (*n* = 108)	RRT group (*n* = 115)	*P* value
6-month mortality	191 (85.7)	98 (90.7)	93 (80.9)	**0.04**
CPC at 6 months				**0.01**
CPC 1	15 (6.7)	3 (2.8)	12 (10.4)	
CPC 2	1 (0.4)	0 (0.0)	1 (0.9)	
CPC 3	4 (1.8)	1 (0.9)	3 (2.6)	
CPC 4	12 (5.4)	6 (5.6)	6 (5.2)	
CPC 5	191 (85.7)	98 (90.7)	93 (80.9)	

Values are expressed as number (%) or median (interquartile range)

*P* < 0.05 are presented in bold

*CPC* cerebral performance category, *RRT* renal replacement therapy

### Descriptive data

AKI was defined in 590/1063 (55.5%) of OHCA patients treated with TTM. Most AKI developed within 3 days of ROSC (461/590 [78.1%]). The frequency of AKI stage was 257/590 [44.5%], 110/590 [18.6%], and 223/590 [37.8%] for stages 1, 2, and 3, respectively. RRT was applied in 115/223 (51.6%) patients with stage 3 AKI. The most common modality among RRT patients was CRRT (111 [96.5%]), and the remaining four patients (3.5%) received intermittent haemodialysis. The average initiation time of RRT was 18 (9–46) h from the time that ROSC and RRT were initiated, which was within 48 h of ROSC in most cases (89/115 [77.4%]). The time to achieve AKI stage 3 in patients with RRT was 1 (1–2) day, and the time to achieve AKI stage 3 in patients without RRT was 3 (2–4) days. Average SCr values from day 1 to 7 following ROSC is provided in the supplementary material (see Additional file 1, Table S1).

### Comparisons of 6-month mortality according to RRT status in different cohorts

**Fig. 2:**
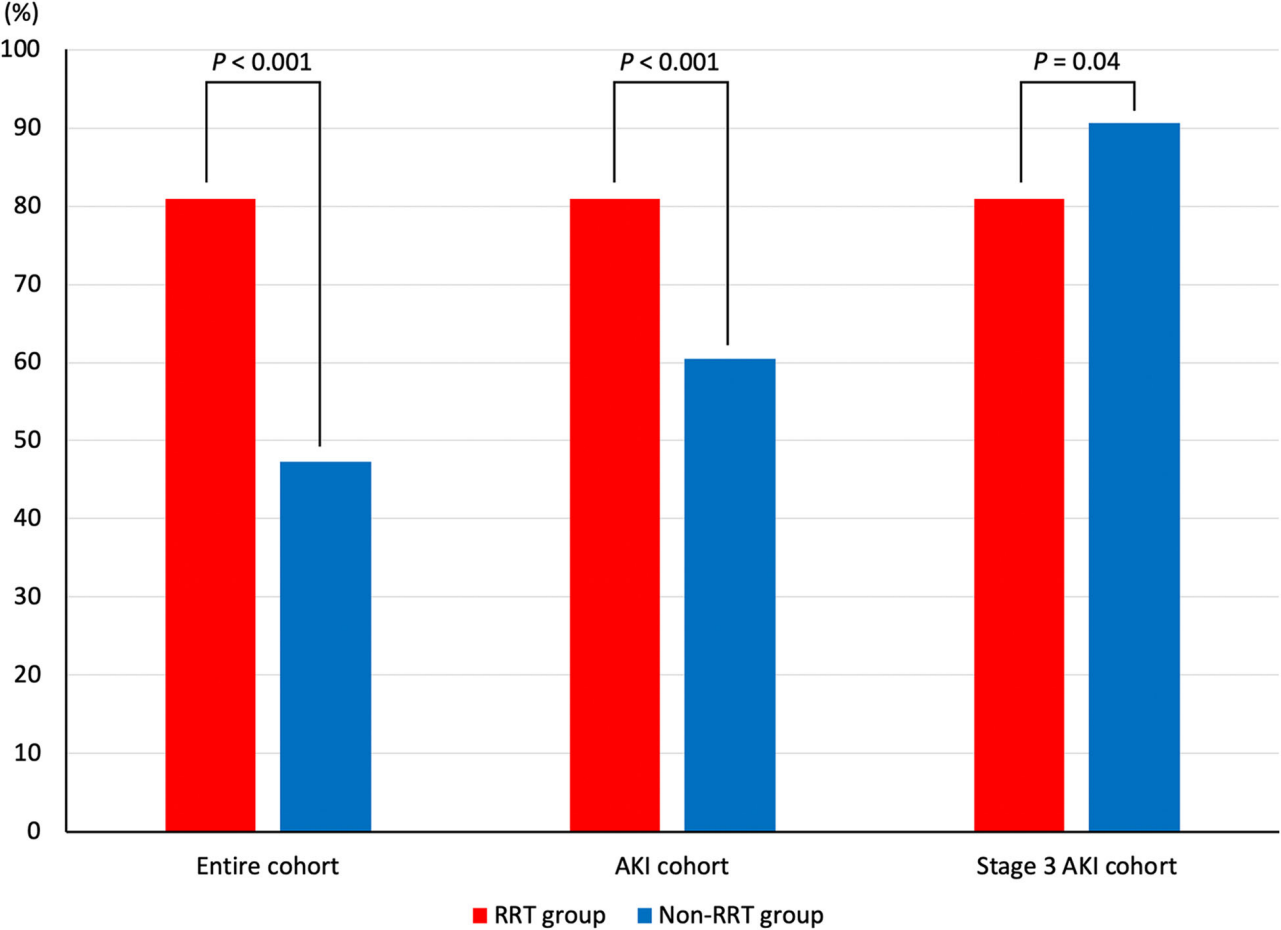
Comparisons of 6-month mortality according to the RRT status in different cohorts. AKI, acute kidney injury; RRT, renal replacement therapy

**Fig. 3:**
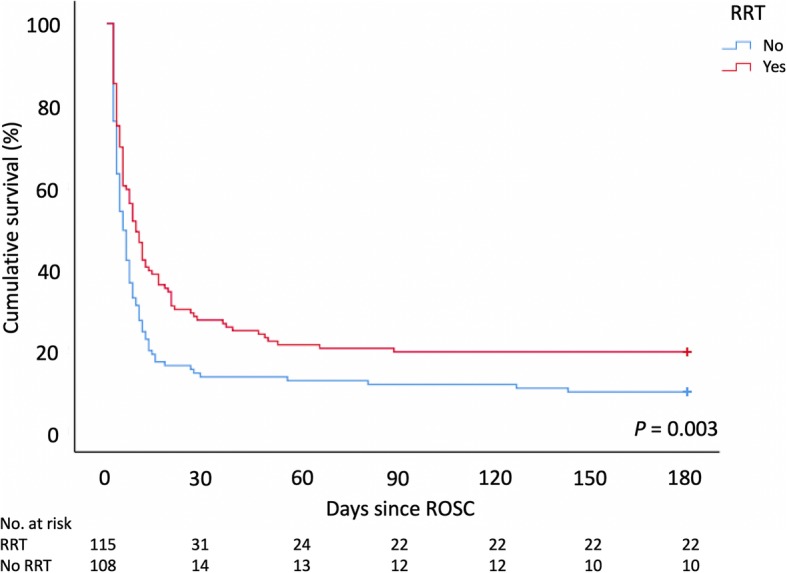
Cumulative survival up to 180 days since return of spontaneous circulation according to renal replacement therapy status. ROSC, return of spontaneous circulation; RRT, renal replacement therapy

The overall 6-month mortality of all enrolled patients (entire cohort, *n* = 1063) was 541/1063 (50.9%). The 6-month mortality of the non-RRT group in the entire cohort was significantly lower than that of the RRT group (448/948 [47.3%] vs. 93/115 [80.9%], *P* < 0.001) (Fig. 2). In case of patients with AKI (AKI cohort, *n* = 590), 6-month mortality was 380/590 (64.4%). The 6-month mortality of the non-RRT group in AKI cohort also was significantly lower than that of the RRT group (287/475 [60.4%] vs. 93/115 [80.9%], *P* < 0.001). However, in patients with stage 3 AKI (stage 3 AKI cohort, *n* = 223), the 6-month mortality of the non-RRT group was significantly higher than that of the RRT group (98/108 [90.7%] vs. 93/115 [80.9%], *P* = 0.04) (Table 2). The Kaplan-Meier survival curves differed significantly between the non-RRT and RRT groups in these cohorts (*P* = 0.003) (Fig. 3).

### Factors associated with 6-month mortality in patients with stage 3 AKI

In the multivariate Cox regression analysis, four variables, namely, body weight, hypoglycaemia, re-arrest, and RRT, were independently associated with risk of death in patients with stage 3 AKI (Table 3). The HR of RRT was 0.569 (95% CI, 0.377–0.857, *P* = 0.01).

**Table 3:** Factors associated with 6-month mortality in patients who developed stage 3 acute kidney injury after an out-of-hospital cardiac arrest

Variable	Coefficient	Standard error	Wald	*P* value	Hazards ratio	95% CI
Age	0.004	0.008	0.271	0.60	1.004	0.989–1.019
Male gender	− 0.033	0.221	0.022	0.88	0.967	0.628–1.491
Body weight	0.021	0.008	7.210	**0.01**	1.021	1.006–1.037
Hypertension	− 0.087	0.213	0.166	0.68	0.917	0.604–1.391
Heart failure	− 1.106	0.569	3.774	0.05	0.331	0.108–1.010
Diabetes mellitus	− 0.078	0.229	0.117	0.73	0.925	0.590–1.448
Arrest location				0.05		
Witnessed arrest	− 0.299	0.233	1.654	0.20	0.741	0.470–1.170
Bystander CPR	− 0.219	0.268	0.669	0.41	0.803	0.475–1.359
Time from collapse to CPR	0.002	0.019	0.015	0.90	1.002	0.965–1.041
Time from CPR to ROSC	− 0.004	0.006	0.342	0.56	0.996	0.984–1.009
Epinephrine, mg	0.043	0.026	2.807	0.09	1.044	0.993–1.098
Initial rhythm				0.42		
Arrest cause				0.56		
Lactate after ROSC	0.034	0.022	2.535	0.11	1.035	0.992–1.080
Shock after ROSC	0.252	0.374	0.453	0.50	1.286	0.618–2.676
TT of 36 °C	− 0.222	0.250	0.789	0.37	0.801	0.490–1.308
TTM duration of 48 h	0.461	0.714	0.417	0.52	1.586	0.392–6.421
^†^ Seizure	− 0.472	0.251	3.526	0.06	0.624	0.381–1.021
^†^ Bleeding	0.333	0.364	0.838	0.36	1.395	0.684–2.845
^†^ Sepsis	0.264	0.228	1.351	0.25	1.303	0.834–2.035
^†^ Hypoglycaemia	0.462	0.230	4.038	**0.04**	1.587	1.011–2.489
^†^ Sustained hyperglycaemia	0.257	0.206	1.561	0.21	1.294	0.864–1.937
^†^ Tachycardia	− 0.236	0.235	1.013	0.31	0.790	0.498–1.251
^†^ Bradycardia	0.069	0.684	0.010	0.92	1.071	0.281–4.089
^†^ Re-arrest	0.767	0.238	10.354	**0.001**	2.152	1.349–3.433
RRT	− 0.565	0.209	7.289	**0.01**	0.569	0.377–0.857

*P* < 0.05 are presented in bold

*CPR* cardiopulmonary resuscitation, *ROSC* return of spontaneous circulation, *RRT* renal replacement therapy, *TT* target temperature, *TTM* targeted temperature management

^†^Event recorded within 7 days since return of spontaneous circulation

### Neurological outcomes according to the RRT status in patients with stage 3 AKI

The distributions of CPC (1–5) at 6 months for the non-RRT vs. RRT groups were 3/108 (2.8%) vs. 12/115 (10.4%) for CPC 1, 0/108 (0.0%) vs. 1/115 (0.9%) for CPC 2, 1/108 (0.9%) vs. 3/115 (2.6%) for CPC 3, 6/108 (5.6%) vs. 6/115 (5.2%) for CPC 4, and 98/108 (90.7%) vs. 93/115 (80.9%) for CPC 5, respectively (*P* = 0.01) (Table 2).

### Reasons for initiating and terminating RRT

Oliguria or anuria was the leading cause of initiating RRT (62/115 [53.9%]). Other causes followed acidosis (30/115 [26.1%]), azotaemia (9/115 [7.8%]), volume overload (8/115 [7.0%]), and hyperkalaemia (6/115 [5.2%]). Reasons for terminating RRT included death (67/115 [58.3%]), recovery from AKI (20/115 [17.4%]), withdrawal of RRT continuation by patient’s legal surrogates (18/115 [15.7%]), and discharge/transfer of the patient (10/115 [8.7%]).

### Dialysis dependence in patients applying RRT

Only 32 RRT patients survived to discharge (32/115 [27.8%]). Among them, 8/32 (25%) still needed RRT at discharge.

## Discussion

Until recently, there was limited evidence on the use of RRT in OHCA patients. Although AKI frequently occurs after OHCA, the recent guidelines did not comment on the role of RRT during PCAS because of insufficient data [1–8, 14, 15]. In this multicentre cohort of severe AKI after OHCA treated with TTM in South Korea, we found that the 6-month mortality of the RRT group was significantly lower than that of the non-RRT group. Moreover, dialysis intervention was associated with lower 6-month mortality in the multivariate Cox regression analysis.

In previous studies, the mortality of the RRT group was not lower than that of the non-RRT group [3, 4, 10, 11]. Winther-Jensen et al. reported that the 30-day mortality of a RRT group was significantly higher than that of a non-RRT group in the cohort of OHCA patients [11]. However, Beitland et al. reported that the 6-month mortality of a RRT group was not different from that of a non-RRT group in the cohort of patients with AKI after OHCA [4], and Geri et al. reported that there were no differences between the RRT and non-RRT groups in a cohort of patients with stage 3 AKI [3]. Considering those results, we compared the mortality between the non-RRT and RRT groups in the different cohorts (all enrolled patients, patients with AKI, and stage 3 patients with AKI). Interestingly, although the 6-month mortality of the RRT group was significantly higher than that of the non-RRT group in the cohorts of all enrolled patients and patients with AKI, the result was reversed in the cohort of patients with stage 3 AKI (Fig. 2). This might be a promising result for nephrologists and intensive care practitioners and implies the need for RRT in patents with stage 3 AKI after OHCA treated with TTM.

However, many factors should be considered in evaluating the results of the current study. First, there were differences in the baseline characteristics of the study cohorts between the present study and previous ones (e.g., age, male sex, witnessed arrest, bystander CPR, time from CPR to ROSC, and shock after ROSC) [3, 4, 10, 11]. Second, there are insufficient data to determine absolute indications and the optimal timing for initiation of RRT. Moreover, some clinicians tend to delay RRT when they suspect that patients may recover on their own and because of well-known risks of RRT itself, including hypotension, arrhythmia, membrane bio-incompatibility, vascular access, and anti-coagulant administration [16]. As a result, the initiation of RRT is extremely variable and based primarily on empiricism and local institutional practice and resources [17]. Therefore, it will be difficult to determine whether to provide RRT based only on whether the patient developed stage 3 AKI after OHCA. The AKI Network stated that the indications for RRT must be viewed within the context of the patient’s entire clinical condition with most indications being relative; there are only a small number of absolute indications [17]. Third, the 6-month mortality of those in the RRT group with stage 3 AKI was extremely high in the present study. Moreover, a longer CPR time, lower ratio of shockable rhythm, and higher ratio of shock may underlie the high mortality in our cohort. In addition, a longer CPR time and lower ratio of shockable rhythm may underlie the higher ratio of shock in our cohort. Therefore, a large-scale multinational, multicentre study or randomised controlled trial will be needed to confirm the exact effect of RRT on the mortality after OHCA because of extremely high mortality in those with stage 3 AKI.

In case of critically ill patients, the overall in-hospital mortality in patients with AKI on dialysis consistently decreased, and the mortality of those with AKI on dialysis was relatively lower than that of the RRT group with OHCA; this is because outcomes associated with OHCA remain poor [18].

Contrary to the present study, some previous studies reported that the RRT was a risk factor for mortality even after correction for disease severity in case of critically ill patients, and the mortality of patients receiving RRT increased in the long-term follow-up over an 8-year period [19, 20]. Differences in disease severity, limits to defining disease severity accurately, and variability in selecting RRT could have contributed to the different results. Additional data from well-designed observational studies will be needed to clarify the long-term mortality of RRT group after OHCA.

Dialysis interventions were initiated within 48 h of ROSC in most cases, and the most common modality was CRRT. This may have been due to the high rate of shock after ROSC. These results are compatible with current standards because CRRT is recommended as the initial RRT modality [21]. A higher frequency of bleeding in the RRT group may have been caused by complications during the insertion of the dual-lumen haemodialysis catheter (Table 1). The ratio of dialysis dependence in our cohort was similar to that in a cohort of critically ill patients [20].

The distribution of CPC was significantly different according to the status of RRT (Table 2). Although only 32 patients with stage 3 AKI survived over 6 months, the high ratio of CPC 1 in the RRT group might suggest a beneficial effect of RRT. Further studies will be needed to verify the role of RRT on neurological outcomes after OHCA.

Although old age and initial non-shockable rhythm were risk factors for developing AKI after OHCA, age and differences in the initial rhythm between the non-RRT and RRT groups were not associated with 6-month mortality in the multivariate Cox regression analysis [1]. Instead, body weight, hypoglycaemia/re-arrest event within 7 days since ROSC, and RRT were associated with 6-month mortality. Interestingly, it was previously reported that overweight was associated with higher survival rate and a better neurological outcome after cardiac arrest [22]. Overweight per se might act protectively after cardiac arrest because of a better nutritive state compared with that of underweight. However, obesity is a well-known risk factor for AKI and mortality in critical illness [23]. As a result, an increase in body weight could act as a risk factor in our cohort of stage 3 AKI. Hypoglycaemia and re-arrest events were associated with poor outcome after cardiac arrest [24, 25]. Therefore, our results are compatible with previous studies.

### Limitations

The present study has several limitations. First, because we defined AKI using only SCr criteria, it is possible that we underestimated AKI prevalence in our cohort. In the case of critically ill patients, the production of SCr decreases. Additionally, a decrease in SCr could be potentiated by therapeutic hypothermia [26]. Therefore, an exact judgement on AKI status is difficult in the case of individuals treated with therapeutic hypothermia. In addition, differences in urine output and fluid balance are likely to have influenced the decision to start RRT. However, data on urine output at RRT initiation and fluid balance could not be evaluated in our cohort. Second, the present study was a retrospective analysis of prospectively collected observational data that did not include information on standardised treatments or management protocols for RRT. Therefore, the risk of selection bias could have been introduced. Third, the initiation of RRT in our study was at the discretion of the physician responsible for the patient’s care according to the KDIGO guideline (e.g., severe hyperkalaemia, severe acidosis, pulmonary oedema, and uremic complication). There were no consistent protocols dictating when to initiate RRT, which might have led to inconsistencies in practices among the principal investigators of each institution. A randomised controlled trial of RRT on patients with stage 3 AKI is needed to confirm the role of RRT. Fourth, there was a possibility of failure to conduct RRT even if urgent RRT was indicated because of the decision on withdrawal of life-sustaining therapy according to the neurological prognostication. However, in South Korea, the withdrawal of life-sustaining therapy depending on the neurological prognostication has not been used widely because of several reasons. For instance, the life-sustaining treatment decisions act was only applied recently in South Korea (since February 2018). In addition, legal surrogates, including family members, could not determine withdrawal of life-sustaining therapy, especially stopping the ventilator or removing the endotracheal tube owing to traditional sentiment. Therefore, we expect that decisions regarding withdrawal of life-sustaining therapy may not affect the initiation of RRT. However, there was a possibility that RRT may be stopped when a poor neurological outcome or death was expected through the treatment course, as the cost for RRT (especially continuous RRT) is quite expensive in South Korea (approximately €400 per day). Fifth, in the cohort of RRT (*n* = 115), 80 patients were diagnosed as achieving stage 3 AKI by initiating RRT itself (Group A) and 35 patients were diagnosed as achieving stage 3 AKI based on SCr (Group B). Although the initiation of RRT was not determined by conditions other than SCr in most cases, daily SCr of Group B was significantly higher than that of Group A (see Additional file 1, Table S2). Therefore, it may be possible to compare patients reaching the AKI stage 3 by non-creatinine criteria with patients reaching the AKI stage 3 by creatinine criteria. The sixth limitation is that the results of the present study may have been affected by the characteristics of our study cohort. Therefore, the results might not be reproducible in another cohort with different characteristics. Fifth, although we excluded the patients who died within 48 h from the time of ROSC initiation to decrease problems related to competing risk, excluding these patients itself may have introduced bias.

## Conclusions

Dialysis interventions were independently associated with a lower risk of death in patients with stage 3 AKI treated with TTM after OHCA.

## Supplementary information

Additional file 1: Supplementary material. **Table S1.** Average serum creatinine values from days 1 to 7 according to the cohort. **Table S2.** Comparisons of daily serum creatinine values between patients who were diagnosed with stage 3 acute kidney injury by initiating renal replacement therapy itself (Group A) and patients who were diagnosed with stage 3 acute kidney injury by serum creatinine value (Group B) in patients undergoing renal replacement therapy.

## Data Availability

The datasets used and/or analysed during the current study are available from the corresponding author on reasonable request.

## Abbreviations

AKI: Acute kidney disease
CI: Confidence interval
CPC: Cerebral performance category
CPR: Cardiopulmonary resuscitation
CRRT: Continuous renal replacement therapy
GFR: Glomerular filtration rate
HR: Hazard ratio
KDIGO: Kidney Disease: Improving Global Outcomes
KORHN: Korean Hypothermia Network
KORHN-PRO: Korean Hypothermia Network prospective registry
OHCA: Out-of-hospital cardiac arrest
PCAS: Post-cardiac arrest syndrome
ROSC: Return of spontaneous circulation
RRT: Renal replacement therapy
SCr: Serum creatinine
TTM: Targeted temperature management
