# Supplementary material for: Renal replacement therapy is independently associated with a lower risk of death in patients with severe acute kidney injury treated with targeted temperature management after out-of-hospital cardiac arrest
Source: Crit Care. 2020 Mar 23;24:115. doi: 10.1186/s13054-020-2822-x (PMC7092437; doi:10.1186/s13054-020-2822-x)
Supplement: Supplementary file 1 — Supplementary material. Table S1. Average serum creatinine values from days 1 to 7 according to the cohort. Table S2. Comparisons of daily serum creatinine values between patients who were diagnosed with stage 3 acute kidney injury by initiating renal replacement therapy itself (Group A) and patients who were diagnosed with stage 3 acute kidney injury by serum creatinine value (Group B) in patients undergoing renal replacement therapy. [file 13054_2020_2822_MOESM1_ESM.docx]

**Supplementary material**

Table S1. Average serum creatinine values from days 1 to 7 according to the cohort

| Sub-cohort | Day 1 SCr (mg/dl) | Day 2 SCr (mg/dl) | Day 3 SCr (mg/dl) | Day 4 SCr (mg/dl) | Day 5 SCr (mg/dl) | Day 6 SCr (mg/dl) | Day 7 SCr (mg/dl) |
| --- | --- | --- | --- | --- | --- | --- | --- |
| Entire cohort | 1.2 (1.0, 1.5) | 0.9 (0.7, 1.3) | 0.9 (0.7, 1.4) | 0.9 (0.7, 1.4) | 0.9 (0.6, 1.3) | 0.8 (0.6, 1.2) | 0.8 (0.6, 1.2) |
| AKI cohort | 1.3 (1.1, 1.7) | 1.2 (0.8, 1.9) | 1.3 (0.9, 2.0) | 1.3 (0.9, 2.1) | 1.2 (0.9, 2.0) | 1.2 (0.9, 1.9) | 1.2 (0.9, 1.9) |
| AKI stage 3 cohort | 1.7 (1.4, 2.3) | 2.2 (1.7, 2.7) | 2.5 (1.6, 3.5) | 2.6 (1.9, 4.0) | 2.6 (1.7, 4.4) | 2.7 (1.6, 4.4) | 2.7 (1.7, 4.4) |
| RRT group | 1.7 (1.5, 2.3) | 2.1 (1.6, 2.8) | 2.2 (1.4, 3.2) | 2.2 (1.7, 3.2) | 2.2 (1.6, 3.4) | 2.0 (1.4, 3.5) | 2.0 (1.5, 3.2) |

Values are expressed as median (interquartile range).

AKI, acute kidney injury; RRT, renal replacement therapy; SCr, serum creatinine

Table S2. Comparisons of daily serum creatinine values between patients who were diagnosed with stage 3 acute kidney injury by initiating renal replacement therapy itself (Group A) and patients who were diagnosed with stage 3 acute kidney injury by serum creatinine value (Group B) in patients undergoing renal replacement therapy

| SCr since ROSC | Group A | Group B | *P*-value |
| --- | --- | --- | --- |
| SCr in day 1 | 1.7 (1.5-2.1) | 2.1 (1.6-3.0) | **<0.001** |
| SCr in day 2 | 1.7 (1.4-2.2) | 2.9 (2.6-3.8) | **<0.001** |
| SCr in day 3 | 1.8 (1.3-2.2) | 3.7 (2.9-4.5) | **<0.001** |
| SCr in day 4 | 1.9 (1.3-2.3) | 3.8 (2.3-5.1) | **<0.001** |
| SCr in day 5 | 1.8 (1.4-2.4) | 3.6 (2.0-5.7) | **<0.001** |
| SCr in day 6 | 1.9 (1.3-2.2) | 3.5 (1.8-4.6) | **0.005** |
| SCr in day 7 | 1.9 (1.3-2.9) | 2.3 (1.8-3.9) | 0.08 |

Values are expressed as median (interquartile range).

*P*<0.05 are presented in bold.

ROSC, return of spontaneous circulation; SCr, serum creatinine
